# Discover the Molecular Biomarker Associated with Cell Death and Extracellular Matrix Module in Ovarian Cancer

**DOI:** 10.1155/2015/735689

**Published:** 2015-03-16

**Authors:** Qiang Liu, Jianxin Guo, Jinghong Cui, Jing Wang, Ping Yi

**Affiliations:** Department of Obstetrics and Gynecology, Research Institute of Surgery, Daping Hospital, Third Military Medical University, Chongqing 40042, China

## Abstract

High throughput technologies have provided many new research methods for ovarian cancer investigation. In tradition, in order to find the underlying functional mechanisms of the survival-associated genes, gene sets enrichment analysis (GSEA) is always regarded as the important choice. However, GSEA produces too many candidate genes and cannot discover the signaling transduction cascades. In this work, we have used a network-based strategy to optimize the discovery of biomarkers using multifactorial data, including patient expression, clinical survival, and protein-protein interaction (PPI) data. The biomarkers discovered by this strategy belong to the network-based biomarker, which is apt to reveal the underlying functional mechanisms of the biomarker. In this work, over 400 expression arrays in ovarian cancer have been analyzed: the results showed that cell death and extracellular module are the main themes related to ovarian cancer progression.

## 1. Introduction

Among women in the United States, ovarian cancer is the eighth most common cancer and the fifth leading cause of cancer death, after lung and bronchus, breast, colorectal, and pancreatic cancers. Ovarian cancer causes more deaths than any other cancers of the female reproductive system. In 2012 ovarian cancer occurred in 239,000 women and resulted in 152,000 deaths. The overall five-year survival rate in the United States is 45%; outcomes are worse in the developing world. To date, the treatment for ovarian cancer mainly involves chemotherapy, surgery, and sometimes radiotherapy. Unfortunately, these adjuvant therapies have only a modest impact on survival time. This situation indicates that development of sensitive diagnostic biomarker used in the early stage of ovarian cancer will greatly lead to improved survival of patients.

Recent researches showed that ovarian cancer may be a heterogeneity disease and multiple signaling pathways contribute to ovarian cancer progression [1–3]. Therefore, functional subnetwork that these genes interconnect may lead to a more precise set of alterations, which could become key network-based survival-associated biomarkers or drug targets for clinical interrogation. In this work, we have combined multifactorial data to identify network-based biomarkers. First, the correlation between the expression of survival-associated gene groups and survival data was quantified by a multivariate Cox proportional hazards model; then, the protein-protein interaction (PPI) network was used to preselect the gene groups obtained by the first step. Hence, we can obtain the network-based survival-associated biomarkers by this approach and with enough biological understanding of molecular mechanism. Over 400 ovarian cancer expression arrays have been analyzed, and extracellular matrix and cell death module were regarded as the main themes associated with ovarian survival data. By manual reading the references, we found that the two subnetworks we obtained contained several previously implicated genes with clinical significance.

## 2. Materials and Methods

### 2.1. Gene Expression and Clinical Dataset and the Protein-Protein Interaction

The gene expression data and the corresponding clinical data were downloaded from The Cancer Genome Atlas (TCGA) database. The gene expression profiling from 562 ovarian patients was measured by UNC_AgilentG4502A_07_3 microarray platform. The sample only with expression profiling but without clinical data is also removed from our analysis. Finally, we get 438 samples having both expression profiling and clinical data, which involves 8972 genes. The protein-protein interactions data from Human Protein Reference Database (HPRD) [4] was used in this study. Currently, HPRD contains manually curated over 42,000 interactions between 9826 human genes.

### 2.2. Survival-Associated Subnetwork

The survival-associated subnetwork was identified by Survnet [5], a webserver for identifying network-based biomarkers that most correlate with patient survival data. The Survnet webserver consists of three component processes: (i) a scoring function (combining the subnetwork property, molecular profile, and patient survival data), (ii) a searching algorithm (for finding the candidate biomarkers), and (iii) an evaluation (validating the statistical significance of the biomarkers). Then, it can search for subnetworks that most correlate with the observed patient survival data. The survival-associated subnetworks produced by Survnet have two kinds of score—network *P* value and multivariate Cox *P* value. Multivariate *P* value was used to identify the survival-associated subnetwork (at *P* < 0.05 level) and the search parameter (search distance) is 2.

### 2.3. Gene Ontology Functional and KEGG Annotations

Cox proportional hazards model was used to correlate each individual gene expression data with survival data (*P* < 0.05). Then, according to these survival-associated genes, the DAVID [6] was used to correlate the survival-associated genes generated with gene ontology (GO) and KEGG functional annotations. False discovery rate at 0.05 level was used to control multiple statistical tests.

### 2.4. MiRNA Analysis

The microRNAs and their target genes were downloaded in miRTarBase [7] (Release 4.5) (an experimentally validated microRNA-target interactions database), which involve 39110 miRNA-target interactions in human. Then, we aimed to discover the miRNAs that significantly regulate the survival-associated module. The gene set enrichment analysis was used to fulfill the aim.

Gene set enrichment analysis (GSEA) is a common statistical technique to reveal the significantly changed modules of the target gene sets against the background to elucidate the underlying functional mechanisms. Here, we use GSEA to discover the miRNAs that significantly regulate the survival-associated network. The score of each miRNA was calculated using the cumulative hypergeometric function as follows:

(1) $$\begin{array}{c} P=\frac{\left(\begin{array}{c} K\\ O \end{array}\right)\left(\begin{array}{c} N-K\\ M-O \end{array}\right)}{\left(\begin{array}{c} N\\ M \end{array}\right)}, \end{array}$$

where the *N* represents the sum of the number of all miRNA-target genes and the number of the module genes. *M* represents the number of the module genes. *K* represents the number of the genes of certain miRNA targets. *O* represents the number of the genes both contained in the module and contained in the miRNA-target genes. With the cutoff of *P* < 0.01, we discover the miRNAs that significantly regulate the module. Multiple statistical tests were controlled by false discovery rate (FDR). All of the above calculations were implemented in R statistical package using function phyper (http://www.r-project.org/). The adjustment of the GSEA score was implemented by R function p.adjust with parameter method = “fdr”.

## 3. Result

### 3.1. Network-Based Survival-Associated Subnetwork

**Figure 1:**
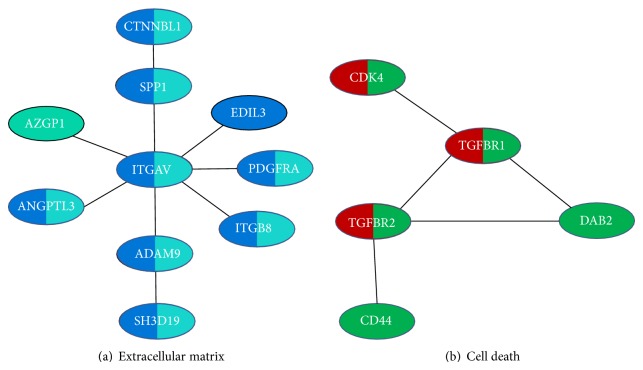
Survival-associated subnetwork in ovarian cancer. (a) Extracellular matrix module and (b) cell death modules. These survival-associated subnetworks are labeled with top 1 in blue, top 2 in red, top 4 in green, and top 5 in cyan. Nodes with more than one color mean that these proteins are involved in more than one survival-associated subnetwork.

In order to test our network-based strategy, we combined human PPI data from HPRD database and a set of gene expression data of 438 ovarian patients with clinical survival data. Among the 9826 nodes (genes) in PPI network, 7486 nodes can be mapped to ovarian cancer gene expression profiling data. We totally get 134 compact survival-associated subnetworks by using Survnet (see Supplementary File 1 in Supplementary Material available online at http://dx.doi.org/10.1155/2015/735689). The top 5 survival-associated subnetworks are summarized in Table 1. These five subnetworks can be classified into two larger functional modules in Figure 1. As shown in Figure 1(a), two survival-associated subnetworks have been associated with cell death module. As shown in Figure 1(b), another two survival-associated subnetworks have been associated with cell death module.

Extracellular matrix module (ECM) consists of ITGAV (integrin, alpha V), ADAM9 (ADAM metallopeptidase domain 9), ANGPTL3 (angiopoietin-like 3), AZGP1 (alpha-2-glycoprotein 1, zinc-binding), EDIL3 (EGF-like repeats and discoidin I-like domains 3), ITGB8 (integrin, beta 8), PDGFRA (platelet-derived growth factor receptor, alpha polypeptide), SPP1 (secreted phosphoprotein 1), SH3D19 (SH3 domain containing 19), and CTNNBL1 (catenin, beta-like 1). The ITGAV-SH3D19 (belonging to the top 1 and 5 subnetwork) forms the integrin-mediated signaling pathway of extracellular matrix module. Integrins are transmembrane receptors that are the bridges for cell-cell and cell-extracellular matrix interactions. When triggered, integrins in turn trigger chemical pathways to the interior (signal transduction), such as the chemical composition and mechanical status of the extracellular matrix module, which results in a response (activation of transcription) such as regulation of the cell cycle, cell shape, and/or motility, or new receptors being added to the cell membrane. Previous studies have demonstrated that integrins and their receptors play a critical role in ovarian cancer progression [8, 9]. As shown in Figure 1(a), ITGAV is the hub of the extracellular matrix module. This gene encodes a protein that is a member of the integrin superfamily. ITGAV interacts with several extracellular matrix proteins to mediate cell adhesion and may play a role in cell migration. It is proposed that this protein may regulate angiogenesis and cancer progression. Previous studies have demonstrated that ITGAV may play a role during progression in a variety of cancers. For example, de Souza Viana et al. have found that overexpression of the ITGAV gene and protein was correlated with an increased risk of perineural invasion [10]. More importantly, ITGAV is connected with ADAM9, ANGPTL3, AZGP1, EDIL3, ITGB8, PDGFRA, and SPP1, which have close relation with cell adhesion, cell communication. It is noted that CTNNBL1 is connected with ITGAV by SPP1; previous studies have proved that CTNNBL1 has a close relation with regulation of apoptosis, regulation of programmed cell death, and membrane-enclosed lumen [11], which implies that the extracellular matrix module may regulate the ovarian cancer progression by regulating apoptosis, main cell death patterns in ovarian cancer cells.

On the other hand, cell death module (CDM) consists of DAB2 (Dab, mitogen-responsive phosphoprotein, homolog 2), TGFBR1 (transforming growth factor, beta receptor 1), TGFBR2 (transforming growth factor, beta receptor II), CDK4 (cyclin-dependent kinase 4), and CD44 (CD44 molecule (Indian blood group)). DAB2 is the hub of this module, which means that DAB2 plays an important role in this module. DAB2 is expressed in normal ovarian epithelial cells but is downregulated or absent from ovarian carcinoma cell lines, suggesting its role as a tumor suppressor [12]. TGFBR1 and TGFBR2 directly interact with DAB2; they encode a member of the Ser/Thr protein kinase family and the TGFB receptor subfamily. The TGFBR1 and TGFBR2 proteins are transmembrane protein that has a protein kinase domain, forms a heterodimeric complex with another receptor protein, and binds TGF-beta. This receptor/ligand complex phosphorylates proteins, which then enter the nucleus and regulate the transcription of a subset of genes related to cell proliferation. Mutations in this gene have been associated with the development of various types of tumors. As for CDK4, it is also known as cell division protein kinase 4. The protein encoded by this gene is a member of the Ser/Thr protein kinase family. This protein is a catalytic subunit of the protein kinase complex that is important for cell cycle G1 phase progression, which is proved to be associated with a variety of cancers [13–15]. Besides, the protein encoded by CD44 is a cell-surface glycoprotein involved in cell-cell interactions and cell adhesion and migration. It is a receptor for hyaluronic acid (HA) and can also interact with other ligands, such as osteopontin, collagens, and matrix metalloproteinases (MMPs). This protein participates in a wide variety of cellular functions including lymphocyte activation, recirculation and homing, hematopoiesis, and tumor metastasis [16]. In sum, all the genes in this module are associated with various cancers and cell death, so DAB2 may regulate ovarian cancer progression by mediating cell death signaling pathway, which may be a novel biomarker in clinical practice.

### 3.2. Survival-Associated Module Enriched by Gene Ontology and KEGG

We also conducted the enrichment analysis of survival-associated genes to GO and KEGG terms. First, using a univariate Cox proportional hazards model, 828 genes were found significantly correlated with survival data (*P* < 0.05). These survival-associated correlated genes were listed in Supplementary Table 2. With a cutoff of FDR < 0.05, we identified 29 cellular component terms, 199 biological process terms, and 22 molecular function terms as well as 3 KEGG pathways that are enriched with survival-associated gene (Supplementary Table 3). The top 5 significant GO terms in each functional category are summarized in Table 2. From the viewpoint of molecular function, the top ranked GO terms include “glycosaminoglycan binding,” “polysaccharide binding,” “pattern binding,” “growth factor binding,” “heparin binding,” and “extracellular matrix binding.” From the viewpoint of cellular component, the top ranked GO terms include “extracellular region part,” “plasma membrane part,” “extracellular region,” “extracellular space,” and “extracellular matrix.” The top ranked KEGG pathways include “Focal adhesion,” “Tight junction,” and “ECM-receptor interaction.” All the above results clearly reflect the molecular changes at extracellular matrix region. On the other hand, the programmed cell death function is dominated in the selected GO biological process categories (Table 2), such as “regulation of cell death,” “regulation of apoptosis.” Therefore, both extracellular matrix and cell death module, the two master themes identified in our network analysis, were reproduced from GO analysis.

Furthermore, our network-based strategy outperforms traditional gene set enrichment analysis. First, each gene set usually includes too many genes with limiting their clinical application. For example, in the above GO enrichment data, there are over 148 and 41 survival genes that are enriched within GO term cell death and extracellular matrix module (Supplementary Table 3). However, in our results based on network-based strategy, there are only 5 and 10 potential biomarker genes in cell death and extracellular matrix module (Figure 1). Besides, interaction relationships between these candidate proteins were also provided by our strategy. Hence, our result will provide direct mechanism understanding of ovarian cancer progression and greatly facilitate further experimental verification.

### 3.3. The miRNAs Regulate the Survival-Associated Module

The GSEA analysis was used to discover miRNAs that significantly regulate the two modules—the extracellular matrix module (ECM) and the cell death module (CDM). The result was listed in Table 3. Li et al. have found that deregulation of miR-128 in ovarian cancer promotes cisplatin resistance, which means that miR-128 may act as a promising therapeutic target for improvement of tumor sensitivity to cisplatin [17]. Besides, Torres et al. have found the miR-26b expressed differentially between malignant and normal tissues [18]. Cao et al. have declared that miR-335 represents an independent prognostic marker in epithelial ovarian cancer [19]. Wang et al. have verified that miR-15b and the other nine miRNAs were identified to be able to distinguish human ovarian cancer tissues from normal tissues with 97% sensitivity and 92% specificity [20]. Imam et al. have declared genomic loss of tumor suppressor miRNA-204 promotes cancer cell migration and invasion by activating AKT/mTOR/Rac1 signaling and actin reorganization [21]. Li et al. have found that the expression of miR-320a is upregulated in the paclitaxel-resistant ST30 cells, which reveals that miR-320a plays an important role in the ovarian cancer progression [22]. In addition, Reimer et al. have revealed that regulation of transcription factor E2F3a by methylating the promoter of miR-34a has a marked relevance with ovarian cancer [23]. It is noted that almost all the miRNAs we found have been verified having a significant relevance with the ovarian cancer, which, on the other hand, reveals that the modules obtained by our method are reliable and has-miR-1 may be a novel therapeutic target.

## 4. Discussion

In recent years, a variety of molecular biomarkers in ovarian cancer have been identified, such as HE4 [24], NPPB [25], and goosecoid homeobox [26]. However, the underlying mechanism of the biomarkers is still unclear that greatly limits the clinical application of the biomarkers. As for the result of the traditional enrichment analysis, the signaling transduction cascades between candidate genes are elusive. Hence, we developed a network-based strategy to identify the survival-associated subnetworks and combine multifactorial data including gene expression data, clinical data, and PPI network. Finally, extracellular matrix and cell death related subnetworks were regarded as survival-associated subnetworks in ovarian cancer, which can reveal the underlying mechanism of the survival-associated genes. Therefore, the biomarkers identified by our strategy are much easier to be applied in clinical therapy.

Our network-based strategy identified two survival-associated modules associated with ovarian cancer progression. The extracellular matrix module plays an important role in cell-cell communication. In ovarian cancer, the module may have an influence on the communication between the malignant cells and the normal cells. Therefore, we speculated that the extracellular matrix module can mediate the ovarian cancer progression by regulating the cancer metastasis. On the other hand, the cell death signaling module is involved in a variety of biological events that include morphogenesis, maintenance of tissue homeostasis, and elimination of harmful cells. Dysfunction of cell death leads to various diseases in humans, especially various cancers [27–29]. In the early stage of the ovarian cancer, the cell death signaling module can restrict cell proliferation by regulating apoptosis and autophagy. While in the advanced stage of the ovarian cancer, the dysfunctional cell death signaling module will facilitate the ovarian cancer progression. Therefore, the cell death signaling module can be a novel network-based biomarker of ovarian cancer.

## Supplementary Material

Supplemental Table 1: 134 compact survival-associated subnetworks by Survnet.
Supplemental Table 2: 828 survival-associated genes by univariate Cox proportional hazards model.
Supplemental Table 3: 29 cellular component terms, 199 biological process terms, 22 molecular function terms and 3 KEGG pathways enriched with survival-associated genes.

## Figures and Tables

**Table 1:** Top 5 survival-associated subnetworks in ovarian cancer.

Network rank	Component genes	Univariate Cox *P* value	Adjusted multivariate Cox *P* value
1	ADAM9	6.82*E* − 03	8.69*E* − 09
	ANGPTL3	1.38*E* − 04	
	CTNNBL1	4.55*E* − 03	
	EDIL3	2.21*E* − 01	
	ITGAV	5.08*E* − 02	
	ITGB8	2.27*E* − 05	
	PDGFRA	5.84*E* − 04	
	SH3D19	5.65*E* − 03	
	SPP1	7.15*E* − 03	

2	APBB2	3.43*E* − 03	1.61*E* − 08
	CDK4	2.31*E* − 03	
	ENOX1	5.48*E* − 03	
	FCHO1	2.18*E* − 01	
	GFI1	1.00*E* − 02	
	KCTD15	9.04*E* − 04	
	RHPN2	5.73*E* − 03	
	RUNX1T1	2.30*E* − 02	
	SMURF1	2.58*E* − 02	
	TGFBR1	6.50*E* − 03	
	TGFBR2	7.10*E* − 04	
	TRIM27	3.56*E* − 03	
	ZBTB16	2.13*E* − 02	

3	ALB	1.09*E* − 02	3.79*E* − 08
	C6orf62	4.13*E* − 01	
	CCDC53	1.02*E* − 02	
	LUC7L2	4.27*E* − 02	
	NDUFA4L2	6.46*E* − 02	
	NSF	1.36*E* − 02	
	PARK2	2.72*E* − 02	
	RAD1	2.00*E* − 03	
	SVIL	2.11*E* − 02	
	UBR1	3.41*E* − 03	

4	ARHGAP17	1.25*E* − 02	4.31*E* − 08
	CD44	3.98*E* − 02	
	CDK4	2.31*E* − 03	
	DAB2	3.22*E* − 03	
	DOCK1	1.04*E* − 01	
	ELMO1	9.51*E* − 02	
	ELMO2	3.75*E* − 02	
	LCK	3.02*E* − 02	
	PACSIN1	9.11*E* − 02	
	RHPN2	5.73*E* − 03	
	RNF5	5.23*E* − 03	
	SH3BP2	3.27*E* − 02	
	TGFBR1	6.50*E* − 03	
	TGFBR2	7.10*E* − 04	
	WASF2	9.98*E* − 02	

5	ADAM9	6.82*E* − 03	9.28*E* − 08
	ANGPTL3	1.38*E* − 04	
	AZGP1	9.12*E* − 01	
	CTNNBL1	4.55*E* − 03	
	ITGAV	5.08*E* − 02	
	ITGB8	2.27*E* − 05	
	PDGFRA	5.84*E* − 04	
	SH3D19	5.65*E* − 03	
	SPP1	7.15*E* − 03	

**Table 2:** Top 5 significant GO terms enriched with survival genes in ovarian cancer.

Rank	Cellular component	FDR	Biological process	FDR	Molecular function	FDR
1	GO:0044421~extracellular region part	1.50*E* − 08	GO:0010941~regulation of cell death	5.32*E* − 32	GO:0005539~glycosaminoglycan binding	4.62*E* − 06
2	GO:0044459~plasma membrane part	3.75*E* − 06	GO:0042981~regulation of apoptosis	2.88*E* − 31	GO:0030247~polysaccharide binding	3.92*E* − 05
3	GO:0005576~extracellular region	3.28*E* − 05	GO:0043067~regulation of programmed cell death	4.99*E* − 31	GO:0001871~pattern binding	3.92*E* − 05
4	GO:0005615~extracellular space	6.77*E* − 05	GO:0007049~cell cycle	5.39*E* − 30	GO:0019838~growth factor binding	1.37*E* − 04
5	GO:0031012~extracellular matrix	5.87*E* − 04	GO:0022402~cell cycle process	5.07*E* − 18	GO:0008201~heparin binding	4.62*E* − 04

**Table 3:** The miRNAs significantly regulate the survival-associated module.

MiRNA symbol	*P* value	Survival module	Target gene
hsa-miR-128-3p	1.86*E* − 14	ECM	AZGP1, SH3D19
hsa-miR-26b-5p	1.29*E* − 07	ECM	ADAM9, PDGFRA
hsa-miR-335-5p	6.45*E* − 06	ECM	ITGB8, SPP1
hsa-miR-1	1.07*E* − 04	CDM	CD44, CDK4
hsa-miR-15b-5p	1.90*E* − 07	CDM	CD44, CDK4
hsa-miR-204-5p	5.73*E* − 09	CDM	TGFBR1, TGFBR2
hsa-miR-320a	1.18*E* − 05	CDM	CD44, CDK4
hsa-miR-335-5p	8.13*E* − 03	CDM	DAB2, TGFBR2
hsa-miR-34a-5p	1.34*E* − 05	CDM	CD44, CDK4
