# Supplementary material for: Discover the Molecular Biomarker Associated with Cell Death and Extracellular Matrix Module in Ovarian Cancer
Source: Biomed Res Int. 2015 Mar 16;2015:735689. doi: 10.1155/2015/735689 (PMC4378326; doi:10.1155/2015/735689)
Supplement: Supplementary file 1 — Supplemental Table 1: 134 compact survival-associated subnetworks by Survnet. Supplemental Table 2: 828 survival-associated genes by univariate Cox proportional hazards model. Supplemental Table 3: 29 cellular component terms, 199 biological process terms, 22 molecular function terms and 3 KEGG pathways enriched with survival-associated genes. [file 735689.f1.zip › Supplement Table 1.pdf]

Rank	Cox *p*-value	FDR	Gene ID	#Nodes	#Edges
1	8.69E-09	3.29E-04	EDIL3	9	8
2	1.61E-08	6.58E-04	FCHO1	13	12
3	3.79E-08	9.87E-04	C6orf62	10	9
4	4.31E-08	1.32E-03	ELMO2	15	16
5	9.28E-08	1.64E-03	AZGP1	9	8
6	1.53E-07	1.97E-03	CYR61	9	8
7	3.24E-07	2.30E-03	CCL16	14	13
8	4.73E-07	2.63E-03	AFP	14	13
9	7.96E-07	2.96E-03	CROCC	12	11
10	1.27E-06	3.29E-03	CLP1	11	10
11	1.67E-06	3.62E-03	IL31RA	12	12
12	2.10E-06	3.95E-03	CCL4	13	12
13	2.20E-06	4.28E-03	CNOT10	9	8
14	2.36E-06	4.61E-03	CTSF	10	11
15	2.51E-06	4.93E-03	SUCLG1	9	8
16	2.52E-06	5.26E-03	RASSF7	9	8
17	2.89E-06	5.59E-03	USH1G	9	8
18	3.18E-06	5.92E-03	CXCR3	12	12
19	3.25E-06	6.25E-03	OPRL1	10	10
20	4.64E-06	6.58E-03	APOD	8	7
21	4.67E-06	6.91E-03	PZP	11	15
22	4.87E-06	7.24E-03	NRD1	14	15
23	5.31E-06	7.57E-03	ANKS4B	9	8
24	5.93E-06	7.89E-03	HLA-DPB1	10	10
25	8.07E-06	8.22E-03	MMP10	10	14
26	9.60E-06	8.55E-03	MMP25	11	11
27	1.03E-05	8.88E-03	C12orf35	14	13
28	1.07E-05	9.21E-03	SPDEF	11	11
29	1.07E-05	9.54E-03	RNASEL	11	11
30	1.14E-05	9.87E-03	HHIP	6	5
31	1.31E-05	1.02E-02	CXCL1	11	15
32	1.39E-05	1.05E-02	SEMA4A	11	10
33	1.73E-05	1.09E-02	RBP1	13	13
34	1.78E-05	1.12E-02	CXCL5	10	14
35	2.26E-05	1.15E-02	THBS2	10	14
36	2.32E-05	1.18E-02	MMP26	11	15
37	2.48E-05	1.22E-02	OMP	11	10
38	2.79E-05	1.25E-02	SLC16A1	9	8
39	3.12E-05	1.28E-02	RECK	10	14
40	3.39E-05	1.32E-02	RAB32	13	13
41	3.42E-05	1.35E-02	MMP12	10	9
42	4.26E-05	1.38E-02	KISS1	11	15
43	4.43E-05	1.41E-02	TIMM8A	11	11
44	4.51E-05	1.45E-02	PTCH2	6	5
45	4.57E-05	1.48E-02	SEC11A	9	8
46	5.09E-05	1.51E-02	IGLL1	11	11
47	5.53E-05	1.55E-02	PON1	13	13

48	6.56E-05	1.58E-02	TRIM3	11	10
49	7.78E-05	1.61E-02	CCL2	14	13
50	8.65E-05	1.64E-02	SRPX2	12	12
51	8.93E-05	1.68E-02	RIC8B	10	9
52	9.65E-05	1.71E-02	OSTM1	11	11
53	9.86E-05	1.74E-02	CCL17	10	10
54	1.04E-04	1.78E-02	KIAA0232	9	8
55	1.12E-04	1.81E-02	PPIL1	10	10
56	1.13E-04	1.84E-02	MOCS2	9	8
57	1.16E-04	1.88E-02	LANCL1	8	7
58	1.31E-04	1.91E-02	MPO	11	14
59	1.85E-04	1.94E-02	HAGH	12	12
60	1.95E-04	1.97E-02	SLC22A11	13	12
61	1.95E-04	2.01E-02	ADAMTS5	12	16
62	2.15E-04	2.04E-02	PABPN1	11	11
63	2.15E-04	2.07E-02	MAEA	12	12
64	2.23E-04	2.11E-02	TKT	12	12
65	2.51E-04	2.14E-02	PTGDR	10	9
66	2.88E-04	2.17E-02	ADAM2	11	11
67	2.94E-04	2.20E-02	SCG3	11	14
68	3.30E-04	2.24E-02	CBX7	10	10
69	3.60E-04	2.27E-02	BSDC1	8	8
70	4.54E-04	2.30E-02	PROC	14	14
71	5.47E-04	2.34E-02	INHBB	15	16
72	8.61E-04	2.37E-02	VHLL	15	14
73	9.00E-04	2.40E-02	SAA4	10	12
74	1.15E-03	2.43E-02	SFTPA1	14	14
75	1.21E-03	2.47E-02	TMEM67	5	4
76	1.37E-03	2.50E-02	PCYT2	2	1
77	1.53E-03	2.53E-02	PNOC	7	7
78	1.55E-03	2.57E-02	F2RL3	14	14
79	1.70E-03	2.60E-02	TFR2	11	10
80	1.71E-03	2.63E-02	CLGN	7	8
81	1.76E-03	2.66E-02	LMBR1L	12	14
82	1.85E-03	2.70E-02	HIST1H1C	11	10
83	1.88E-03	2.73E-02	RGS17	6	5
84	1.88E-03	2.76E-02	PHEX	9	8
85	2.34E-03	2.80E-02	CX3CL1	2	1
86	2.56E-03	2.83E-02	CBLN3	3	2
87	2.72E-03	2.86E-02	HABP2	9	9
88	2.75E-03	2.89E-02	CPN2	10	9
89	2.78E-03	2.93E-02	KAL1	10	12
90	2.85E-03	2.96E-02	RXFP4	9	11
91	2.93E-03	2.99E-02	THBD	14	15
92	3.00E-03	3.03E-02	MICB	7	6
93	3.33E-03	3.06E-02	BSN	13	12

94	3.45E-03	3.09E-02	POLK	12	12
95	3.46E-03	3.13E-02	FZD6	8	8
96	3.55E-03	3.16E-02	CRYBA1	6	5
97	3.66E-03	3.19E-02	SERPINE2	10	12
98	4.93E-03	3.22E-02	F13B	8	11
99	5.19E-03	3.26E-02	WNT6	9	8
100	5.92E-03	3.29E-02	SFTPD	10	12
101	6.58E-03	3.32E-02	REG1A	10	12
102	8.08E-03	3.36E-02	RAET1G	8	7
103	8.64E-03	3.39E-02	NPNT	12	15
104	8.69E-03	3.42E-02	COL6A3	11	14
105	8.95E-03	3.45E-02	XCL1	11	12
106	9.34E-03	3.49E-02	CCL20	11	12
107	9.61E-03	3.52E-02	CHID1	2	1
108	9.90E-03	3.55E-02	P4HA3	9	11
109	1.03E-02	3.59E-02	GNRH1	10	12
110	1.06E-02	3.62E-02	HPN	10	12
111	1.28E-02	3.65E-02	IL10	15	19
112	1.30E-02	3.68E-02	CFHR1	11	12
113	1.31E-02	3.72E-02	COL6A1	9	9
114	1.38E-02	3.75E-02	TRHDE	11	13
115	1.41E-02	3.78E-02	PRPF18	4	3
116	1.41E-02	3.82E-02	GALNT6	9	11
117	1.44E-02	3.85E-02	PCOLCE	9	9
118	1.66E-02	3.88E-02	INHBE	10	12
119	1.93E-02	3.91E-02	THBS3	9	12
120	1.98E-02	3.95E-02	SELP	11	12
121	2.12E-02	3.98E-02	TTC35	2	1
122	2.37E-02	4.01E-02	KISS1R	10	13
123	2.44E-02	4.05E-02	SPINK1	9	11
124	2.81E-02	4.08E-02	AMY2A	3	2
125	3.08E-02	4.11E-02	PPP3R2	2	1
126	3.16E-02	4.14E-02	COL5A3	9	11
127	3.19E-02	4.18E-02	CXCL2	11	12
128	3.41E-02	4.21E-02	SAA1	13	15
129	3.76E-02	4.24E-02	PRKD2	9	8
130	3.92E-02	4.28E-02	GRPR	6	5
131	4.05E-02	4.31E-02	WISP1	8	10
132	4.05E-02	4.34E-02	PLA2R1	9	11
133	4.41E-02	4.38E-02	LUM	9	11
134	4.73E-02	4.41E-02	TNFAIP6	9	11
