# Supplementary material for: Discover the Molecular Biomarker Associated with Cell Death and Extracellular Matrix Module in Ovarian Cancer
Source: Biomed Res Int. 2015 Mar 16;2015:735689. doi: 10.1155/2015/735689 (PMC4378326; doi:10.1155/2015/735689)
Supplement: Supplementary file 1 — Supplemental Table 1: 134 compact survival-associated subnetworks by Survnet. Supplemental Table 2: 828 survival-associated genes by univariate Cox proportional hazards model. Supplemental Table 3: 29 cellular component terms, 199 biological process terms, 22 molecular function terms and 3 KEGG pathways enriched with survival-associated genes. [file 735689.f1.zip › Supplement Table 2.pdf]

Gene Symb	Cox Score
GALNT10	9.99E-07
AVPR1B	5.62E-06
TLL1	1.87E-05
ITGB8	2.27E-05
RPS6KA2	3.83E-05
MMRN1	5.17E-05
RNASE2	1.19E-04
ANGPTL3	1.38E-04
PALB2	1.49E-04
C5AR1	1.74E-04
GAS1	2.80E-04
FZD1	2.84E-04
HABP2	3.16E-04
EIF3K	3.26E-04
MYL2	3.52E-04
PPP3CA	3.63E-04
WIPF2	3.98E-04
DOM3Z	4.36E-04
SNX24	4.36E-04
QKI	4.37E-04
RAB11FIP5	4.41E-04
P2RY12	5.22E-04
PDGFRA	5.84E-04
CX3CR1	6.17E-04
KCNA1	6.50E-04
SHMT2	6.94E-04
TGFBR2	7.10E-04
PRLR	7.37E-04
HNF4A	7.40E-04
CALB2	7.65E-04
CBLN4	8.41E-04
SAMD4B	8.81E-04
KCTD15	9.04E-04
KCTD13	1.00E-03
GRIA1	1.02E-03
QPRT	1.02E-03
ITSN1	1.02E-03
PHKA1	1.07E-03
IGFBP4	1.10E-03
MYH1	1.13E-03
HYI	1.18E-03
WISP1	1.23E-03
CLEC5A	1.29E-03
APC	1.37E-03
PTGER3	1.38E-03
KIF22	1.38E-03
RAB31	1.40E-03
MARCKSL1	1.53E-03
F13B	1.64E-03

MSR1	1.72E-03
MED1	1.77E-03
ESM1	1.79E-03
HSPB7	1.82E-03
XRCC6BP1	1.94E-03
EFEMP1	1.96E-03
GALNTL2	1.97E-03
DKK2	1.98E-03
IL31RA	1.99E-03
RAD1	2.00E-03
RGS4	2.07E-03
NRG4	2.12E-03
AEBP2	2.12E-03
IGSF21	2.13E-03
SELP	2.16E-03
PDIA4	2.17E-03
PABPN1	2.19E-03
MYPN	2.22E-03
FGF5	2.26E-03
PDGFD	2.26E-03
TEAD1	2.28E-03
CDK4	2.31E-03
CACNA1G	2.32E-03
CD93	2.33E-03
WDR77	2.38E-03
KIF3A	2.43E-03
NFKBIB	2.43E-03
ECH1	2.47E-03
ZNF521	2.47E-03
LMOD1	2.56E-03
STAB1	2.60E-03
NSUN4	2.67E-03
INSM1	2.69E-03
VSTM2L	2.70E-03
CSNK1G3	2.70E-03
ASAH1	2.70E-03
MTMR8	2.72E-03
STK3	2.91E-03
PDIA5	2.91E-03
HNF4G	2.99E-03
CACNA1C	3.03E-03
TGFBI	3.05E-03
OLR1	3.08E-03
PEX3	3.11E-03
RABGEF1	3.16E-03
BLOC1S1	3.18E-03
TUFM	3.20E-03
DAB2	3.22E-03
EGR2	3.24E-03
B4GALT3	3.25E-03

BCL7A	3.27E-03
PSMD8	3.36E-03
ZFP36	3.40E-03
UBR1	3.41E-03
APBB2	3.43E-03
TRIM27	3.56E-03
MED29	3.57E-03
SDPR	3.57E-03
UBE2J1	3.62E-03
AGXT	3.63E-03
CASP2	3.68E-03
CNTN1	3.73E-03
CATSPER1	3.79E-03
MYO5A	3.89E-03
CCL15	3.97E-03
JMJD5	4.03E-03
APEX2	4.13E-03
PPFIBP1	4.23E-03
ZNF239	4.35E-03
ANXA4	4.40E-03
RNF8	4.43E-03
PROCR	4.55E-03
CTNNBL1	4.55E-03
BICD2	4.63E-03
GNAI1	4.69E-03
SLC25A10	4.74E-03
FZD5	4.77E-03
CLTCL1	4.81E-03
LSM4	4.88E-03
C1orf35	4.90E-03
COL5A2	4.92E-03
STAC2	4.97E-03
ZNRD1	5.08E-03
HTR2B	5.11E-03
RNF5	5.23E-03
APOD	5.33E-03
ZNF365	5.39E-03
ENOX1	5.48E-03
RRAD	5.54E-03
SH3D19	5.65E-03
THPO	5.68E-03
RHPN2	5.73E-03
ZFP36L1	5.73E-03
ZNF408	5.74E-03
LBP	5.77E-03
MAF	5.77E-03
IL18R1	5.80E-03
SLC22A3	5.82E-03
NEDD4	5.88E-03
PMF1	5.92E-03

GAD1	5.94E-03
WWP1	5.96E-03
PTRF	5.96E-03
FGF7	5.97E-03
KCND2	5.97E-03
PSG3	6.00E-03
RELL1	6.07E-03
SLA2	6.07E-03
BNIP2	6.11E-03
NEXN	6.18E-03
NUAK1	6.24E-03
SIRPA	6.30E-03
MYL1	6.31E-03
RIN2	6.32E-03
PLXNB3	6.38E-03
TGFBR1	6.50E-03
MCM3	6.53E-03
TMOD2	6.62E-03
PRKG1	6.67E-03
XRCC4	6.67E-03
YIPF3	6.69E-03
PDLIM3	6.69E-03
DDX20	6.73E-03
SNRPA1	6.74E-03
TNRC4	6.77E-03
ADAM9	6.82E-03
SYT4	6.85E-03
IL1R2	7.02E-03
CSF1	7.07E-03
GNG2	7.08E-03
LINGO1	7.09E-03
PI3	7.11E-03
AQP1	7.14E-03
SPP1	7.15E-03
FN1	7.22E-03
SNX22	7.23E-03
RPS16	7.25E-03
FGA	7.29E-03
MGP	7.37E-03
AEBP1	7.46E-03
LIPC	7.49E-03
PRR14	7.57E-03
NUP160	7.67E-03
PUF60	7.70E-03
NLRP12	7.70E-03
SHE	7.86E-03
ZFP106	7.89E-03
DLD	7.97E-03
FAM46C	7.99E-03
DCN	8.07E-03

RAD50	8.19E-03
KATNAL1	8.26E-03
USP9Y	8.27E-03
MAPKBP1	8.35E-03
THBS1	8.42E-03
SPDEF	8.51E-03
EZH1	8.55E-03
PHLDB2	8.67E-03
TREM2	8.71E-03
CSF2	8.77E-03
TBC1D10C	8.81E-03
DPM3	8.82E-03
SSBP4	8.92E-03
ACTN4	9.01E-03
C8orf48	9.02E-03
RCAN1	9.08E-03
CCDC88B	9.17E-03
RASSF8	9.19E-03
C16orf53	9.22E-03
ARHGAP29	9.24E-03
AP4E1	9.36E-03
GNG12	9.40E-03
NUCB2	9.45E-03
OTUB2	9.47E-03
RHOU	9.52E-03
CSDE1	9.61E-03
FAP	9.62E-03
ST6GAL1	9.64E-03
NBL1	9.64E-03
FCGR2A	9.84E-03
HERC3	9.97E-03
GFI1	1.00E-02
ELK3	1.00E-02
THBS2	1.01E-02
GDPD2	1.01E-02
SOCS5	1.01E-02
CCDC53	1.02E-02
CDC14B	1.02E-02
MRVI1	1.02E-02
RNASEL	1.02E-02
KLHDC5	1.03E-02
RASGRF1	1.04E-02
PTPN13	1.04E-02
FBXL18	1.05E-02
BRP44L	1.05E-02
C8B	1.06E-02
GRLF1	1.06E-02
AFF4	1.06E-02
ZNF295	1.06E-02
ABCF1	1.09E-02

ALB	1.09E-02
COL8A2	1.10E-02
CYR61	1.12E-02
CBX8	1.12E-02
COL14A1	1.13E-02
ZNF689	1.14E-02
ANXA2	1.15E-02
WWOX	1.16E-02
OVGP1	1.17E-02
WFDC2	1.17E-02
MYH7	1.17E-02
RFX4	1.19E-02
RBBP4	1.19E-02
RDBP	1.20E-02
TXNL4B	1.20E-02
EID3	1.21E-02
UTY	1.21E-02
DYX1C1	1.22E-02
HBG2	1.23E-02
VANGL1	1.23E-02
COL1A2	1.23E-02
PJA2	1.25E-02
HNRNPC	1.25E-02
ARHGAP17	1.25E-02
BANF1	1.26E-02
COX4NB	1.27E-02
BAT5	1.28E-02
IL1RL2	1.30E-02
SSRP1	1.30E-02
IRAK1BP1	1.31E-02
MEA1	1.33E-02
HGF	1.33E-02
HUS1B	1.35E-02
TOE1	1.35E-02
NEBL	1.35E-02
ERBB2	1.36E-02
NSF	1.36E-02
PCOLCE	1.38E-02
FRK	1.39E-02
BRD2	1.39E-02
GRIA3	1.40E-02
HTRA1	1.40E-02
NKX2-2	1.40E-02
GALNT5	1.40E-02
AFAP1	1.41E-02
IL12A	1.41E-02
PDILT	1.42E-02
TTN	1.42E-02
COL8A1	1.42E-02
PSG1	1.43E-02

TJP1	1.43E-02
EIF3J	1.44E-02
TFB1M	1.44E-02
FSTL3	1.44E-02
TOM1L2	1.46E-02
SUZ12	1.46E-02
GRAP2	1.47E-02
LY9	1.47E-02
MEOX2	1.47E-02
CUGBP1	1.49E-02
AP3S1	1.49E-02
MTMR9	1.49E-02
HOXB3	1.50E-02
KRAS	1.51E-02
AKAP12	1.52E-02
METTL1	1.52E-02
CBX5	1.53E-02
TRIT1	1.53E-02
GALNT6	1.55E-02
STK39	1.56E-02
NLGN4X	1.56E-02
SLC7A11	1.56E-02
PALLD	1.57E-02
TREM1	1.58E-02
SCMH1	1.58E-02
PARP2	1.58E-02
SFRP2	1.59E-02
ZHX3	1.60E-02
WDR91	1.60E-02
EHD2	1.62E-02
PSMB5	1.63E-02
ARHGAP19	1.63E-02
FBXO30	1.63E-02
COL11A1	1.64E-02
CD38	1.65E-02
VPS4B	1.66E-02
FOXP4	1.66E-02
ABCB8	1.66E-02
FLNC	1.66E-02
PHGDH	1.67E-02
GTF3C1	1.68E-02
MED19	1.68E-02
WNT7B	1.68E-02
PMPCB	1.69E-02
PAK4	1.70E-02
TOMM20	1.70E-02
ZSCAN16	1.70E-02
MOG	1.71E-02
COPZ2	1.71E-02
TACR1	1.71E-02

C7orf16	1.72E-02
SNRPC	1.72E-02
ALPL	1.74E-02
FANCA	1.74E-02
CD36	1.74E-02
APEX1	1.74E-02
CTGF	1.74E-02
CCDC127	1.74E-02
TFPI2	1.75E-02
PLN	1.75E-02
GDF11	1.75E-02
HOXB4	1.75E-02
PTPRK	1.76E-02
OLFML3	1.77E-02
MBL2	1.78E-02
CRLS1	1.78E-02
PRPS1	1.78E-02
CDH11	1.79E-02
TGFB1I1	1.79E-02
P4HA2	1.80E-02
SLC18A2	1.80E-02
COL6A1	1.81E-02
NR1H3	1.81E-02
PPARA	1.81E-02
IRF4	1.82E-02
HOPX	1.82E-02
LATS2	1.82E-02
PRDX5	1.83E-02
RGS18	1.84E-02
CCL11	1.85E-02
NUFIP2	1.85E-02
SRPK3	1.85E-02
SMARCB1	1.85E-02
PPP2R5D	1.85E-02
RAC3	1.85E-02
RXRG	1.86E-02
HMBS	1.87E-02
PLK2	1.87E-02
SERPINA10	1.87E-02
PTPN1	1.87E-02
MYO9A	1.89E-02
NUCB1	1.89E-02
GRB7	1.90E-02
FCER1A	1.90E-02
RUNX1	1.90E-02
RECQL	1.90E-02
NRK	1.92E-02
SH2D4A	1.92E-02
SH2B1	1.92E-02
NMU	1.93E-02

PPIC	1.94E-02
MMP12	1.94E-02
LCMT1	1.95E-02
CTSA	1.95E-02
ISL2	1.95E-02
TARBP2	1.96E-02
GPR1	1.97E-02
COL5A1	1.99E-02
ZNF76	2.01E-02
WDR12	2.01E-02
PTPRD	2.02E-02
EPB41L3	2.02E-02
SPARCL1	2.02E-02
RINT1	2.04E-02
EFNA4	2.04E-02
MS4A7	2.06E-02
PMP22	2.08E-02
ADAM2	2.10E-02
AGTR1	2.10E-02
EMILIN1	2.11E-02
SVIL	2.11E-02
RGPD5	2.12E-02
CRYAB	2.12E-02
GDF6	2.13E-02
ZBTB16	2.13E-02
SDCBP2	2.13E-02
NTRK2	2.14E-02
FAM127A	2.15E-02
CSNK1A1L	2.15E-02
SIM1	2.16E-02
SCN4B	2.16E-02
NIPSNAP1	2.16E-02
TMEM67	2.16E-02
UNC5CL	2.17E-02
TASP1	2.17E-02
MYOM2	2.18E-02
PAXIP1	2.19E-02
EPHA4	2.19E-02
HOXB2	2.20E-02
SIX3	2.20E-02
CSNK1G1	2.21E-02
CBLN1	2.21E-02
PLAUR	2.21E-02
KRR1	2.23E-02
ORC2L	2.23E-02
SVEP1	2.26E-02
TDP1	2.26E-02
FZD3	2.27E-02
ANTXR1	2.27E-02
TRPM7	2.28E-02

MEF2C	2.28E-02
SNAI1	2.28E-02
RUNX1T1	2.30E-02
FABP4	2.30E-02
DAGLB	2.30E-02
PDPN	2.30E-02
SPOCK1	2.31E-02
PPP1R13L	2.33E-02
PTPN11	2.33E-02
COPS8	2.35E-02
ITGAM	2.36E-02
PLXNC1	2.36E-02
MEF2A	2.36E-02
SRPRB	2.37E-02
GMNN	2.37E-02
CRYBA4	2.38E-02
STAC3	2.39E-02
PEX6	2.40E-02
RNF183	2.41E-02
DYNLRB1	2.41E-02
WARS	2.41E-02
MASP1	2.43E-02
FAM131A	2.44E-02
DSG3	2.44E-02
JAM2	2.44E-02
CEL	2.45E-02
EXOC6B	2.47E-02
KLF13	2.47E-02
PAX3	2.49E-02
C18orf56	2.49E-02
C20orf3	2.50E-02
PDPK1	2.50E-02
RNF7	2.50E-02
SENP2	2.51E-02
ACOT7	2.51E-02
GINS2	2.51E-02
CLDN6	2.51E-02
GALT	2.53E-02
CETP	2.54E-02
KCNB1	2.55E-02
KIRREL	2.57E-02
SMURF1	2.58E-02
NR5A1	2.58E-02
FHL5	2.59E-02
GAS2	2.59E-02
MYO18B	2.60E-02
POLA2	2.64E-02
PACSIN3	2.65E-02
KIF13A	2.65E-02
BYSL	2.66E-02

BSDC1	2.67E-02
PIP5K1B	2.68E-02
TNFRSF13E	2.68E-02
IGFBP6	2.69E-02
SHPRH	2.69E-02
POLD3	2.69E-02
KRIT1	2.70E-02
AK5	2.71E-02
CCL17	2.71E-02
EPB41L2	2.72E-02
ADI1	2.72E-02
HIRIP3	2.72E-02
RP2	2.72E-02
PARK2	2.72E-02
RFX1	2.72E-02
F11	2.73E-02
SLC6A1	2.73E-02
CDC5L	2.74E-02
ACSL3	2.75E-02
HSBP1	2.75E-02
CREB3L3	2.76E-02
EPS8	2.76E-02
PRUNE2	2.77E-02
KLF2	2.77E-02
CRB2	2.78E-02
FGF1	2.79E-02
DMC1	2.79E-02
HIC1	2.79E-02
EDNRA	2.81E-02
TEK	2.81E-02
DPT	2.81E-02
RYR1	2.82E-02
MMP9	2.82E-02
VGLL1	2.83E-02
LUM	2.83E-02
C3AR1	2.84E-02
DARC	2.85E-02
SCT	2.85E-02
SAPS2	2.86E-02
CHD6	2.86E-02
PDE10A	2.86E-02
EHMT2	2.87E-02
PHF23	2.88E-02
GEM	2.89E-02
TUBGCP4	2.89E-02
TRIB1	2.89E-02
BRSK2	2.89E-02
SH3PXD2A	2.89E-02
MKS1	2.90E-02
SSR4	2.90E-02

COL3A1	2.90E-02
PARD6B	2.90E-02
KNG1	2.91E-02
CELSR1	2.95E-02
SEC22B	2.95E-02
IFT20	2.96E-02
THOC4	2.97E-02
CITED4	2.97E-02
AGR2	2.98E-02
MYO16	2.98E-02
PKD2	2.99E-02
VEGFA	3.00E-02
KCND1	3.00E-02
SCLT1	3.01E-02
TCP1	3.01E-02
PLXNA1	3.02E-02
EIF1	3.02E-02
LCK	3.02E-02
ZNF426	3.03E-02
PSMB3	3.03E-02
SH2B3	3.04E-02
MTA2	3.04E-02
GJB2	3.06E-02
ZNFX1	3.06E-02
P4HA3	3.07E-02
SLC2A2	3.08E-02
FAM124B	3.09E-02
SIAH2	3.09E-02
GJB1	3.10E-02
ARL8B	3.10E-02
GADD45B	3.11E-02
ARHGEF2	3.12E-02
TIMP3	3.12E-02
ITGB1	3.13E-02
GRP	3.15E-02
CDH7	3.15E-02
VIP	3.15E-02
SLC4A8	3.15E-02
SRCAP	3.15E-02
FRMD6	3.16E-02
EID1	3.17E-02
SLIT2	3.21E-02
EPB41L1	3.21E-02
PKNOX1	3.23E-02
CCBP2	3.23E-02
CDKN1B	3.25E-02
SH3BP2	3.27E-02
MAP3K2	3.28E-02
FANCG	3.29E-02
PLEKHF2	3.30E-02

TTC28	3.30E-02
LRRFIP1	3.30E-02
SLC1A2	3.31E-02
CLDN11	3.31E-02
MEP1A	3.32E-02
CBX4	3.32E-02
PDK1	3.33E-02
PDHB	3.34E-02
MMP25	3.35E-02
RXRB	3.37E-02
ALDOB	3.38E-02
PATZ1	3.38E-02
ZCCHC17	3.41E-02
EFNA3	3.41E-02
FKBPL	3.42E-02
MDK	3.42E-02
RBM39	3.43E-02
UNC5C	3.44E-02
COL25A1	3.46E-02
CBX7	3.47E-02
TNFRSF8	3.47E-02
IKBKG	3.48E-02
PPAP2A	3.48E-02
TSSK6	3.48E-02
AKAP11	3.49E-02
STOM	3.49E-02
HERC1	3.49E-02
EID2	3.49E-02
CD86	3.50E-02
MUC7	3.50E-02
DYNLT3	3.51E-02
ZNF764	3.52E-02
RETN	3.52E-02
RECK	3.52E-02
WISP2	3.53E-02
TRPV4	3.55E-02
RAD52	3.55E-02
APH1A	3.56E-02
MLNR	3.57E-02
STARD13	3.58E-02
MS4A1	3.58E-02
PPIL1	3.58E-02
F13A1	3.59E-02
ERN1	3.60E-02
TCF21	3.60E-02
SIGLEC1	3.60E-02
BEX2	3.62E-02
PLA2R1	3.63E-02
RGR	3.63E-02
CD200R1	3.63E-02

VPS24	3.64E-02
SEC24B	3.66E-02
BMP4	3.68E-02
TIE1	3.68E-02
IKBKAP	3.69E-02
UBLCP1	3.71E-02
RPL12	3.72E-02
MYH2	3.72E-02
ZBTB5	3.73E-02
UACA	3.74E-02
PAAF1	3.74E-02
CCHCR1	3.74E-02
ELMO2	3.75E-02
CUL3	3.76E-02
POLR2G	3.76E-02
PTPRO	3.78E-02
REPS2	3.78E-02
VCAN	3.78E-02
HTR7	3.80E-02
PPP1R3A	3.81E-02
KLF6	3.82E-02
RBBP6	3.82E-02
UBD	3.84E-02
BFSP1	3.85E-02
SYNCRIP	3.87E-02
KEL	3.87E-02
RAPGEF6	3.89E-02
PCSK5	3.89E-02
DUSP1	3.89E-02
PAK1IP1	3.90E-02
USP8	3.90E-02
OSM	3.90E-02
CAST	3.90E-02
WNT4	3.90E-02
FLOT2	3.91E-02
NAV1	3.91E-02
PRRX1	3.93E-02
AGTPBP1	3.94E-02
RALGPS1	3.95E-02
ZNF609	3.97E-02
CCL1	3.97E-02
CD44	3.98E-02
MLLT6	3.98E-02
CD3E	3.99E-02
PPIH	3.99E-02
ATXN2L	4.00E-02
HABP4	4.00E-02
ELN	4.01E-02
JUN	4.01E-02
FNBP4	4.02E-02

ODF2L	4.03E-02
KIAA1217	4.03E-02
ELF5	4.03E-02
MCF2	4.03E-02
TREML1	4.04E-02
RASSF2	4.05E-02
KAL1	4.06E-02
NIF3L1	4.06E-02
FRS3	4.06E-02
PDLIM5	4.07E-02
PDE4DIP	4.08E-02
RAB13	4.08E-02
SNRPA	4.10E-02
DRD3	4.10E-02
LSM2	4.11E-02
RAI14	4.11E-02
DNAJB2	4.12E-02
TSHR	4.12E-02
ILF2	4.13E-02
SLC4A2	4.15E-02
CCDC101	4.16E-02
SLC22A9	4.17E-02
FOXO1	4.17E-02
IL24	4.17E-02
PSME3	4.18E-02
DCP2	4.18E-02
HLA-DOB	4.19E-02
ADAM12	4.20E-02
GTF2H4	4.22E-02
FANCF	4.22E-02
FOSB	4.24E-02
CNN1	4.25E-02
LUC7L2	4.27E-02
ADRA2A	4.27E-02
GNG10	4.28E-02
HOXA11	4.28E-02
HOOK3	4.29E-02
CDSN	4.29E-02
RAB34	4.29E-02
LSAMP	4.30E-02
LTA4H	4.30E-02
TSPAN6	4.32E-02
GPS1	4.33E-02
CALD1	4.34E-02
CSNK1D	4.34E-02
FAM69B	4.36E-02
C16orf45	4.37E-02
GABRB1	4.38E-02
APOH	4.40E-02
ICAM3	4.41E-02

EFNA5	4.41E-02
CD79A	4.41E-02
CRYBB2	4.42E-02
ITGA5	4.44E-02
MRPS12	4.44E-02
PODXL2	4.45E-02
NEK6	4.45E-02
DLGAP4	4.46E-02
TEF	4.46E-02
LTA	4.47E-02
PAICS	4.48E-02
SLC26A8	4.49E-02
MAPRE1	4.50E-02
PEX19	4.50E-02
LDB2	4.52E-02
RRM2B	4.52E-02
SNCA	4.53E-02
PRPF3	4.53E-02
NVL	4.54E-02
ELP3	4.54E-02
C10orf119	4.54E-02
TARS	4.54E-02
NR2F1	4.54E-02
SART3	4.54E-02
PSMA5	4.56E-02
SNF8	4.57E-02
SNRK	4.57E-02
BCHE	4.59E-02
UTP6	4.59E-02
APOC2	4.60E-02
SPARC	4.61E-02
ISYNA1	4.61E-02
PHKG2	4.62E-02
MIF	4.62E-02
ARHGAP21	4.62E-02
KCNH5	4.65E-02
ARFGEF1	4.67E-02
SCAMP1	4.68E-02
JUNB	4.69E-02
MLLT4	4.69E-02
SLC8A1	4.69E-02
COL6A2	4.70E-02
C17orf70	4.70E-02
TDH	4.70E-02
CEACAM3	4.73E-02
SSBP1	4.76E-02
FASTK	4.76E-02
RGS2	4.76E-02
AMHR2	4.76E-02
PLEKHB1	4.78E-02

IMPA1	4.79E-02
DKC1	4.80E-02
MARCKS	4.81E-02
ITGB6	4.82E-02
CD33	4.82E-02
EMCN	4.84E-02
RAB5B	4.86E-02
CXCR4	4.86E-02
TNFSF11	4.87E-02
TRIM23	4.88E-02
RB1	4.90E-02
SYNJ1	4.92E-02
MLH3	4.93E-02
GOLM1	4.94E-02
PDE3B	4.94E-02
CLEC3B	4.94E-02
ANKRD13A	4.94E-02
TSHB	4.94E-02
TACC1	4.95E-02
HOXC8	4.95E-02
EFCAB6	4.96E-02
CAB39	4.96E-02
PSG9	4.97E-02
UPK2	4.98E-02
SRPK1	4.98E-02
PLA2G5	4.99E-02
FBLN1	4.99E-02
WDR7	4.99E-02
ATXN7L2	5.00E-02
