# Supplementary material for: Discover the Molecular Biomarker Associated with Cell Death and Extracellular Matrix Module in Ovarian Cancer
Source: Biomed Res Int. 2015 Mar 16;2015:735689. doi: 10.1155/2015/735689 (PMC4378326; doi:10.1155/2015/735689)
Supplement: Supplementary file 1 — Supplemental Table 1: 134 compact survival-associated subnetworks by Survnet. Supplemental Table 2: 828 survival-associated genes by univariate Cox proportional hazards model. Supplemental Table 3: 29 cellular component terms, 199 biological process terms, 22 molecular function terms and 3 KEGG pathways enriched with survival-associated genes. [file 735689.f1.zip › supplement Table 3.pdf]

Category	Term	Count	%	PValue	Genes
KEGG_PATHWAY	hsa04510:F	29	3.51E+00	3.75E-05	MYL2, ERBI
KEGG_PATHWAY	hsa04512:E	17	2.06E+00	4.50E-05	COL3A1, C(
KEGG_PATHWAY	hsa04530:T	18	2.18E+00	3.59E-03	PARD6B, M

List Total	Pop Hits	Pop Total	Fold Enrichı	Bonferroni	Benjamini	FDR
317	201	5085	2.31E+00	5.90E-03	5.90E-03	4.51E-02
317	84	5085	3.25E+00	7.08E-03	3.55E-03	5.41E-02
317	134	5085	2.15E+00	4.33E-01	1.72E-01	4.24E+00
